# Discovery of T Cell Antigens by High-Throughput Screening of Synthetic Minigene Libraries

**DOI:** 10.1371/journal.pone.0029949

**Published:** 2012-01-12

**Authors:** Brian D. Hondowicz, Katharine V. Schwedhelm, Arnold Kas, Michael A. Tasch, Crystal Rawlings, Nirasha Ramchurren, Martin McIntosh, Leonard A. D'Amico, Srinath Sanda, Nathan E. Standifer, Jay Shendure, Brad Stone

**Affiliations:** 1 Translational Research Program, Benaroya Research Institute, Seattle, Washington, United States of America; 2 Clinical Immunology, Amgen, Seattle, Washington, United States of America; 3 Department of Genome Sciences, University of Washington, Seattle, Washington, United States of America; 4 Computational Biology, Fred Hutchinson Cancer Research Center, Seattle, Washington, United States of America; University of Cape Town, South Africa

## Abstract

The identification of novel T cell antigens is central to basic and translational research in autoimmunity, tumor immunology, transplant immunology, and vaccine design for infectious disease. However, current methods for T cell antigen discovery are low throughput, and fail to explore a wide range of potential antigen-receptor interactions. To overcome these limitations, we developed a method in which programmable microarrays are used to cost-effectively synthesize complex libraries of thousands of minigenes that collectively encode the content of hundreds of candidate protein targets. Minigene-derived mRNA are transfected into autologous antigen presenting cells and used to challenge complex populations of purified peripheral blood CD8+ T cells in multiplex, parallel ELISPOT assays. In this proof-of-concept study, we apply synthetic minigene screening to identify two novel pancreatic islet autoantigens targeted in a patient with Type I Diabetes. To our knowledge, this is the first successful screen of a highly complex, synthetic minigene library for identification of a T cell antigen. In principle, responses against the full protein complement of any tissue or pathogen can be assayed by this approach, suggesting that further optimization of synthetic libraries holds promise for high throughput antigen discovery.

## Introduction

The efficient and comprehensive discovery of novel, relevant T cell antigens in human subjects and animal model systems is complicated by two factors. The first challenge is that peripheral blood contains an extremely diverse T cell repertoire, with T cells specific for a single antigen present at frequencies ranging from one in 10^5^ to one in 10^2^ peripheral blood mononuclear cells (PBMC). The potentially large ratio of irrelevant-to-relevant cells means that a modest background response derived from the irrelevant cell population can obscure responses from genuine, but rare antigen specific T cells. The second challenge is that most tissues or pathogens express hundreds or thousands of proteins, each representing a potential T cell target antigen. Such large numbers of potential targets are difficult to express in autologous antigen presenting cells (APC) required for typical screening assays.

The historical approach to these practical difficulties has been to either reduce the complexity of the T cell population being screened, or to reduce the number of candidate antigens being tested. For example, reducing the complexity of the T cell population being screened can be accomplished by arbitrarily cloning individual T cells or creating T cell hybridomas from an antigen responsive population [1]–[3]. Clones are expanded and used as sensitive, homogenous reporters for screening large, complex peptide or cDNA libraries. Large numbers of T cells with a single specificity enables the discovery of rare target antigens within the library. An alternative approach is to select one or a very small number of “candidate antigens”, and test these against complex populations of T cells obtained from peripheral blood or splenocyte preparations. The efficient expression of a single candidate antigen in large numbers of APC allows detection of rare T cell specificities within a mixed population [4]–[6]. Both of these approaches are limited – the former, in that only a small number of T cell specificities are assayed, and the latter, in that only a small number of potential target antigens are tested. Hence, we have developed a novel, high-throughput protocol using synthetic minigene libraries capable of screening mixed populations of CD8+ T cells for responses against hundreds of proteins. As a first test of this technology, we synthesized and screened a library encoding all peptides from 186 genes expressed preferentially in human islets using CD8+ T cells from two subjects newly diagnosed with type I diabetes. These screens have identified two novel T cell epitopes targeted by subjects newly diagnosed with or at risk for type I diabetes.

## Materials and Methods

### 2.1. Ethics statement

This project was reviewed and approved by the Virginia Mason Institutional Review Board, which provides IRB oversight for the Benaroya Research Institute at Virginia Mason and Virginia Mason Hospital. Following IRB approval, specimens were provided to the researchers in a de-identified manner by the Benaroya Research Institute Clinical Core Repository. Virginia Mason Institutional Review Board (IRB) is organized and operates in compliance with the U.S. Department of Health and Human Services and U.S. Food and Drug Administration regulations for the protection of human subjects as described in 45 CFR Parts 46, 160, 164 and 21 CFR Parts 50, 56, 312 and 812 and adheres to the International Conference on Harmonisation (ICH) Good Clinical Practice (GCP) guidelines, as applicable.

### 2.2. Gene selection

Selection of genes exhibiting preferential expression in human islets was performed using custom scripts to evaluate the Novartis GeneAtlas V2 microarray dataset (http://www.ncbi.nlm.nih.gov/geo/) [7]. Several criteria were used to select genes for inclusion in our library. First, all genes previously considered as potential autoantibody targets by Wenzlau and Hutton *et al.* were included [8]. The 68 genes considered by these researchers include the known T1D T-cell and autoantibody target antigens. An additional 111 genes were selected based upon combinations of the percentile score (P), the entropy score (Q) and the number of tissues expressing the gene (N). Specifically, from among the genes not evaluated by Hutton *et al.*, we included: a) all genes with a P-rank in the top 100, b) all genes with the Q-rank in the top 100, c) all genes with N<4 for which the Q-rank is in the top 400, and d) all genes with N<10 and either the P-rank or Q-rank is in the top 200. An additional 7 genes with overlapping expression in islets and glomeruli were added to test for autoimmune responses against both tissues. This approach resulted in a list of 186 candidate autoantigenic gene products. Genes, with associated N-score, P-score and Q-scores are presented in **Table S1**.

### 2.3. Library design

A minigene library was designed by extracting overlapping 33 codon open reading frames (ORFs) covering the entire coding domain of the 186 candidate target genes. Overlaps of 10 codons were included between adjacent minigenes, resulting in a library of 3,670 minigenes (minigene sequences are listed in **Table S2**). Minigenes representing all ns SNPs with a frequency of >20% in common populations were included. Each minigene includes in order, a pool specific primer, a T7 promoter, Kozak start, 33 codon open reading frame (ORF), and a common primer. Unique sense, pool-specific primers were included for groups of 10 minigenes (**Table S2**). Minigenes encoding overlapping coding domains were distributed into separate pools. Antisense templates of all minigenes were synthesized in parallel using programmable microarrays, cleaved from the array and supplied as a single oligonucleotide mixture [9].

### 2.4. Minigene library amplification and in vitro transcription

Libraries (10 pmol) were suspended in 100 ul water with 0.1% Tween-20. Initial amplifications of each minigene pool were carried out in 96-well plates with each well containing a 50 ul reaction volume. Each reaction included 200 nM pool specific primer, 200 nM common-3 primer, 1× Herculase® Hotstart buffer, 200 nM each dNTP, 2 U Herculase® hotstart polymerase and 0.5 *f*mol library (1 ul/well of a 1∶200 library dilution). Minigene pools were amplified using cycling profiles of 1 cycle of 96°C for 1 minute, 30 cycles of 96°C for 30 seconds, 53°C for 30 seconds and 72°C for 1 minute, followed by a single 72°C step for 5 minutes. Eight randomly selected PCR products/plate were evaluated on a 2% agarose gel. In this manner, initial PCR reactions with 370 unique, pool-specific sense primers, each combined individually with the common antisense primer, subdivided the library into ordered, nonredundant arrays of 370 pools with ∼10 minigenes/pool (all primers listed in **Table S2**). The use of antisense templates provides complementary sequences for the pool-specific primers, but not the common primer, thereby limiting amplification to only the subset of minigenes selected by an individual pool-specific primer within a single well.

**Figure 1:**
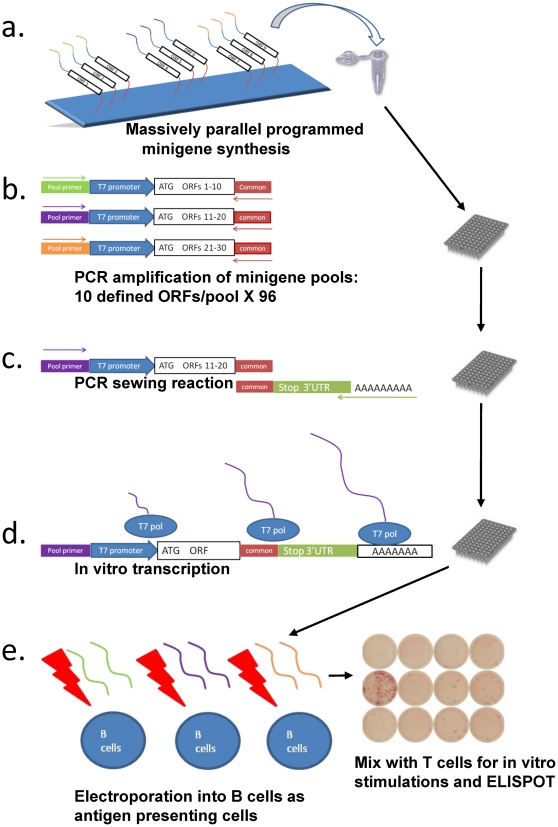
Overview of synthetic minigene screening. **a**) Libraries are synthesized on programmable microarrays, cleaved from the chip surface and provided as a single mixture of antisense oligonucleotide templates. **b**) Initial 96 well PCR reactions utilize individual sense, pool specific primers (green, purple, orange arrows) in combination with a common primer (red arrows) to amplify specific pools of antisense templates (multicolor regions) from the mixed oligo library. Synthesis of a complement for the common primer is dependent upon synthesis of the sense strand primed by a single, unique pool-specific primer in each well. This subdivides the library into ordered arrays of minigene pools, with 10 defined minigenes/well. **c**) A second PCR reaction sews a stop codon and a human beta-globin 3′ UTR (purple+gold boxes) onto each minigene using the common primer domain as an overlap. PCR is driven by the sense pool specific primer, and an antisense primer extending from a 130 base oligo dT tail through the 3′ end of the UTR. Inclusion of an oligo dT tail on the antisense strand encodes a polyA template on the end of each mature minigene. This template allows synthesis of poly-adenylated mRNA during *in vitro* transcription. **d**) Arrays of minigene pools are purified and subject to *in vitro* transcription in the presence of a cap analogue, producing an array of defined, fully translatable mRNA pools. **e**) IVT products are transfected into autologous CD40L expanded B cells for use as antigen presenting cells. Transfected APCs are used as stimulators and targets for *in vitro* stimulations and IFNγ ELISPOT assays.

A second sewing PCR reaction was performed to add a human β-globin 3′UTR and polyA sequence to each minigene using the common primer sequence as the overlap (**Fig. 1**). Inclusion of 130 bases of polyT sequence on the 5′ end of the antisense sewing primer (Ultramer®, Integrated DNA Technologies) eliminates the need to polyadenylate the RNA following *in vitro* transcription. Sewing PCR reactions were also performed in a 96-well format with 50 ul reaction volumes using a cycling profile identical to the initial PCR. Each well contained 4 ul of the initial amplification products, a beta-globin UTR amplification product (∼20 ng/well), 200 nM of the appropriate pool specific primer and 200 nM of the antisense UTR/polyT ultramer. Eight randomly selected PCR products from each plate were evaluated on a 2% agarose gel. PCR products were purified using a Qiagen Minelute^®^ 96-UF PCR purification kit according to the manufacturer's instructions.

Ambion T7 mMessage mMachine^®^ kits were used to produce capped, polyadenylated transcripts from each minigene pool. Reactions were assembled in 96 well plates with a final volume of 20 ul/well using 3 ul of purified minigene pool/reaction as a template. IVT (*in vitro* transcription) plates were incubated for 3 hours at 37°C. Randomly selected IVT products were briefly heated at 65°C and evaluated on a 2% agarose gel. IVT reactions were immediately frozen pending transfections. IVT reactions do not require purification prior to transfection.

### 2.5. T cell and B cell purification

Two newly diagnosed subjects (ND2, ND3, both within 12 months of T1D diagnosis) were provided for screening by the Diabetes Clinical Research Consortium Repository at the Benaroya Research Institute. PBMC from fresh 200 ml blood draws were purified using ficoll density centrifugation and CD8+ T cells were purified using a human CD8+ positive selection kit from Dynal. T cells were immediately frozen in aliquots of 10 and 20×10^6^/cryovial (>97% purity).

Cultures of B cells from each subject were expanded for use as antigen presenting cells (APC) by co-culture of CD8-depleted PBMC on irradiated CD40L expressing L cells [10] in the presence of 10 ng/ml of human IL-21 (Preprotech) and 1.25 µg/ml of Cyclosporin A (Sigma). Expansion cultures were incubated for 2 weeks and non-adherent cells were evaluated by flow cytometric analysis. CD40L and IL-21 stiumulation promoted B cell expansion and the Cyclosporin A prevented T cell proliferation leading to cultures that were >90% CD19+ and DR+.

### 2.6. Transfection (Nucleoporation) of minigene pool IVT products

Nucleoporations were carried out using an AMAXA 96-well shuttle and an AMAXA B cell kit. Each nucleoporation included 2×10^5^ autologous CD40L-expanded B cells resuspended in freshly mixed nucleoporation solution and 3 ul IVT reaction. Nucleoporations were performed using an AMAXA-96 well shuttle and program E101. After nucleoporation the cells were rested for 10 minutes before being placed into pre-warmed tissue culture media. Wells nucleoporated with a GFP encoding plasmid were evaluated by flow cytometric analysis the following day. Transfection efficiencies of >70% GFP expressing, viable cells are commonly achieved.

### 2.7. Minigene-based cultured IFNγ ELISPOT assay

2×10^5^ irradiated (3000 rads), transfected B cells were placed into 96-well round bottom plates with 2×10^4^ autologous CD8**^+^** T cells and 10 ng/mL of rhIL-15 for 7–14 days. Typical survival of B cells following nucleoporation was ∼20% yielding 2–4×10^4^ viable APC/well. After 7–14 days, additional non-irradiated B cells were nucleoporated with the same IVT pools used for *in vitro* stimulations. Each well of stimulated T cells was re-challenged in an *IFNγ* ELISPOT with 2×10^5^ transfected B cells expressing the same minigene pool used for stimulation. ELISPOT plates are incubated for 16–18 hours and developed according to manufacturer's instructions (BD Biosciences).

### 2.8. Peptide-based “direct” IFNγ ELISPOT assay

Direct IFNγ ELISPOT assays do not include an *in vitro* stimulation prior to ELISPOT. The direct IFNγ ELISPOT assay used 1×10^6^ total PBMC+peptide (purchased from Sigma) as both responder and APC populations. ELISPOTs were incubated overnight with 10 µg/ml of the indicated peptides and developed. For assays testing T cell recognition of GLIPR1 (4–12), 1×10^6^ T2 cells were loaded with peptide at room temperature for 2 hours, washed to remove free peptide and mixed with 1×10^5^ CD8+ T cells overnight. Direct ELISPOTs were developed with a slight modification of the protocol used to develop cultured ELISPOT plates. Briefly, following addition of the biotinylated detection antibody and SA-HRP, plates were washed and exposed to a biotinylated anti-avidin D antibody (5 µg/ml) for 1 hour. After washing, plates were exposed to SA-HRP a second time, washed and developed with AEC reagent in the same manner as all previous ELISPOTs.

### 2.9. T2 Binding assay

To determine binding specificity of peptides for HLA-A*0201, 1×10^6^ T2 cells were incubated with 20 µg/ml of the indicated peptide in media for 4 hours at 37°C. Influenza matrix peptide (58–66) was used as a positive control for binding [11] and no peptide was used as the negative control. Cells were washed with PBS and then stained with FITC-anti-HLA-A2 (BB7.2; BDBiosciences) for 20 minutes at 4°C. Surface HLA levels were then assessed on a FACSCalibur and analyzed using Flowjo software.

### 2.10. Statistics

A positive response to an experimental compared to control peptide was determined by ranking log transformed assay results by p-values calculated from moderated t-statistics comparing triplicates of each experimental peptide to triplicates of its matched control peptide using the approach implemented in the R package Limma [12]. P-values were then adjusted for multiple comparisons using the algorithm of Benjamini and Hochberg [13] to calculate false discovery rates (FDR). This approach for ranking and calculating FDR's have been shown to be more conservative than standard permuted t-tests for experiments with small samples [14]. Diagnostic plots (qqnorm; R package) were used to assure that in the average of all control triplicates approximated a normal distribution. Samples with a false discovery rate under 0.05 were considered positive responses.

## Results

### 3.1. Outline of protocol

To increase the efficiency by which the full matrix of potential T cell-antigen interactions can be explored, we developed the novel approach diagrammed in **Figure 1**. Briefly, overlapping minigenes are designed to encode all potential peptide epitopes derived from hundreds of selected proteins expressed in the tissue or pathogen of interest. Long oligonucleotides encoding these minigenes are synthesized in parallel on a programmable microarray, and then released from the array as a single mixture [15] (Fig. 1a). Subsets of minigenes are amplified from this mixture to generate ordered 96-well arrays of minigene pools, with 10 defined minigenes per pool, i.e. ∼960 minigenes per plate (Fig. 1b, c). Each pool is transcribed *in vitro* (IVT) with a cap analogue, thereby synthesizing defined pools of fully translatable mRNAs (Fig. 1d). IVT products from each pool are transfected into irradiated autologous B cells in a 96-well format for use as antigen presenting cells (APC) (Fig. 1e). Transfected minigene-derived mRNAs direct cytoplasmic expression of 33 residue peptides, which are processed and presented by the endogenous MHC class I pathway. Minigene-expressing B cells are utilized to challenge purified CD8+ T cells in a cultured, IFNγ ELISPOT (Fig. 1e). Minigene pools stimulating IFNγ release significantly above controls are scored as positive and subjected to deconvolution assays designed to identify the targeted peptide antigen within a given pool. This approach is effectively a highly multiplexed candidate antigen screen, and allows a large number of potential antigens to be tested against complex mixtures of T cells in a high-throughput fashion.

### 3.2. Screening a minigene library with CD8+ T cells from T1D patients

**Figure 2:**
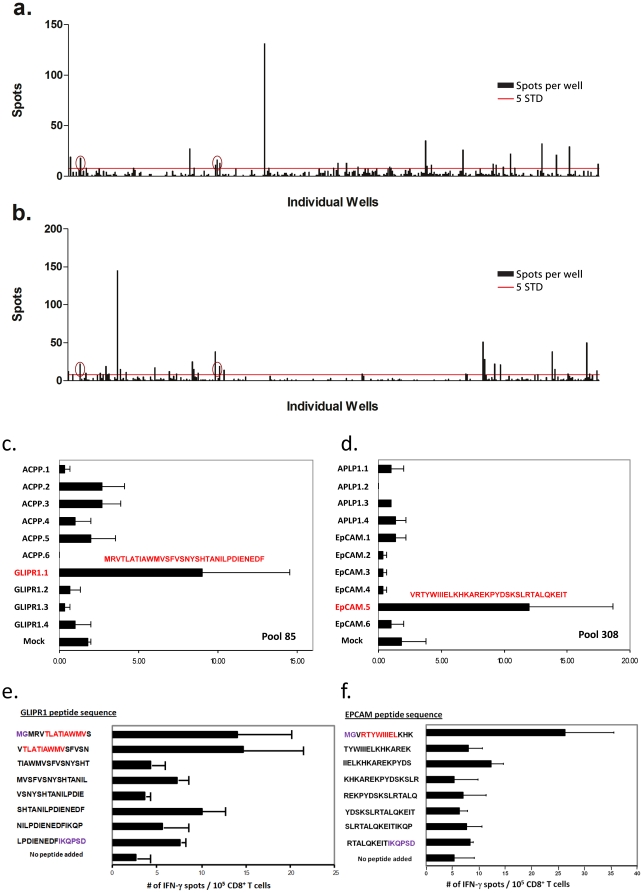
Screening and pool deconvolution ELISPOTs. **Screening: a**) and **b**) are bar graphs of primary screening results from two newly diagnosed T1D subjects. Panel a) is subject ND2, panel b) is subject ND3. Each bar represents the IFNγ spot number for an individual cultured ELISPOT well stimulated with a single minigene pool. Red line indicates 5× standard deviations of wells stimulated with mock transfected autologous B cells. Red circles indicate three wells that scored positive in both screens. **Deconvolution c**) and **d**): cultured IFNγ ELISPOT assays testing ND2-derived CD8+ T cell responses against individual minigenes from **c**) pool 85 and **d**) pool 308. Individual minigenes were amplified using minigene specific primers. Subsequent PCR reactions added T7 and common sequences, and full length individual minigenes were rebuilt and tested as described in Fig. 1. Targeted minigenes encode 33 residue peptides displayed in red. **Peptide epitope mapping e**) and **f**): Direct IFNγ ELISPOT assays testing overlapping 15 residue peptides from GLIPR1 and EpCAM minigenes targeted in b) and c). Peptides were tested in triplicate using a direct 24 hour IFNγ ELISPOT assay with 1×10^5^ CD8+ T cells/well. Purple residues indicate non-antigen derived sequences encoded by minigene flanking sequences. Red residues indicate 9 residue peptide epitope identified in subsequent epitope mapping experiments.

As a proof-of-concept, we constructed a minigene library encoding 186 human pancreatic islet genes. The library was screened with CD8+ T cells from two subjects newly diagnosed with type I diabetes (T1D). Candidate antigens were selected using tissue expression microarrays to identify candidates exhibiting preferential expression in human islets (**Materials and Methods 2.2.**) [8], [16]. The minigene library was designed to encode overlapping 33 residue peptides representing the entire peptide complement of the 186 selected genes and all variations with frequencies of >20% (**Tables S1 & S2**). Amplification and *in vitro* transcription resulted in 370 minigene-derived mRNA pools. Primary screening of the entire library was performed using a cultured IFNγ ELISPOT in which T cells were first stimulated with irradiated APC expressing minigenes from a single pool, cultured for one week to expand T cells responsive to minigene-encoded antigens, and then rechallenged with the same APC/minigene pool in an IFNγ ELISPOT assay (**Fig. 2**, **panels a and b**). These experiments identified 19 and 12 pools stimulating IFNγ release 5 standard deviations above wells stimulated with mock transfected APCs (**Table 1**). Three targeted pools common to both subjects included peptides derived from 4 genes not known to be T1D autoantigens - SCG3, ELL2, PPP1R1A and SERPIN3A. Pools containing minigenes derived from two known T1D autoantigens, CPE and PTPRN/IA2, also scored positive.

**Table 1:** Responses to GLIPR1 4–12 and EpCAM 140–148 incases and controls, and HLA restriction studies.

	ND2>5 STD over mock			ND3>5 STD over mock ND3	
spot #	gene	Pool #l	spot #	gene	Pool #l
131	SORL1	137	145	HPN/TTFG3	35
35	OLFM4/ENO2	249	51	TOB1/PTPRN	289
32	CPE/CUZD1/NPY	330	50	ns SNP pool #361	361
29	WNT4/RAMP2/APOH	349	38	SERPINA3/PPPIR1A	103
27	ACPP/GLIPR1	85	38	PNLIPRP2/PRPH	337
26	SCG2	263	28	PTPRN	290
22	APLP1/EPCAM	308	25	GLIPR1/DNAJC12/STC1	87
21	PRPH/VGF	340	22	SCG3/ELL2	9
19	SYT13/FOXA1	2	22	CLDN7/KCNMB2/ATP2A3	297
18	SCG3/ELL2	9	21	C6	301
16	SERPINA3/PPPIR1A	104	19	PPPIR1A	106
13	PPPIR1A	106	19	INPP5E	27
13	PAPSS2/ELA2A	194			
12	MNX1/CXCL2/CTRB2	296			
12	ns SNP pool # 369	369			
11	SERPINA3/PPPIR1A	103			
11	PROM1	253			
11	CLDN7/KCNMB2/ATP2A3	298			
10	OLFM4/ENO2	250			

Spot number and minigene source for provisionally positive minigene pools. Each score represents a single cultured ELISPOT well stimulated with minigenes derived from the indicated genes. Minigenes in each pool are listed in Table S2. Positive scores exceeded 5 standard deviations of mean scores of wells stimulated with mock transfected autologous B cells. Pool number 9, 103 and 106 scored positive in both subjects.

### 3.3. Deconvolution of positive wells from the primary screen

Individual minigenes within the pools scoring positive in the primary screen were amplified using minigene-specific primers, then rebuilt into full minigenes using stepout primers. Each individual minigene was then retested in triplicate to identify those stimulating a reproducible response. Cultured ELISPOT assays clearly confirmed responses against individual minigenes from two pools targeted by T cells from subject ND2, one derived from epithelial adhesion molecule, EpCAM in pool #308, and another encoding a portion of glioma pathogenesis associated protein, GLIPR1 in pool #85 (**Fig. 2** **, panels c and d**). It is interesting to note that the majority of putatively positive pools from the primary screen did not clearly yield a unique targeted minigene during deconvolution. The reason for this variability remains unknown, but may include low precursor frequencies of T cells specific for individual target epitopes, variable transfection efficiency and/or death rates of transfected APCs, errors within the library, or detection of rare responses against components of the IVT reactions or transfection solution. Nevertheless, even with the apparent high false positive rate we were able to detect two novel epitopes using this technology.

### 3.4. Identifying GLIPR1 and EpCAM epitopes and determining HLA-A2 binding

The two confirmed minigene targets each encoded unique, 33 residue peptides, VRTYWIIIELKHKAREKPYDSKSLRTALQKEIT from EpCAM (139–171, blue residues in **Fig. 2 c**) and MRVTLATIAWMVSFVSNYSHTANILPDIENEDF from GLIPR1 (1–33, red residues in **Fig. 2 c**). To further define the domain containing the minimal peptide epitope, we tested sequential 15 residue peptides with 11 residue overlaps in a direct IFNγ ELISPOT assay. This assay evaluates responses in unselected PBMC from the responding subject *without* an *in vitro* stimulation prior to the ELISPOT assay, and is therefore thought to be a more accurate assessment of ongoing immune reactivity *in vivo*. Two 15 residue GLIPR1 peptides overlapping by 11 residues, and a single 15 residue peptide derived from EpCAM, elicited significant responses (**Fig. 2** **, panels e and f**).

**Figure 3:**
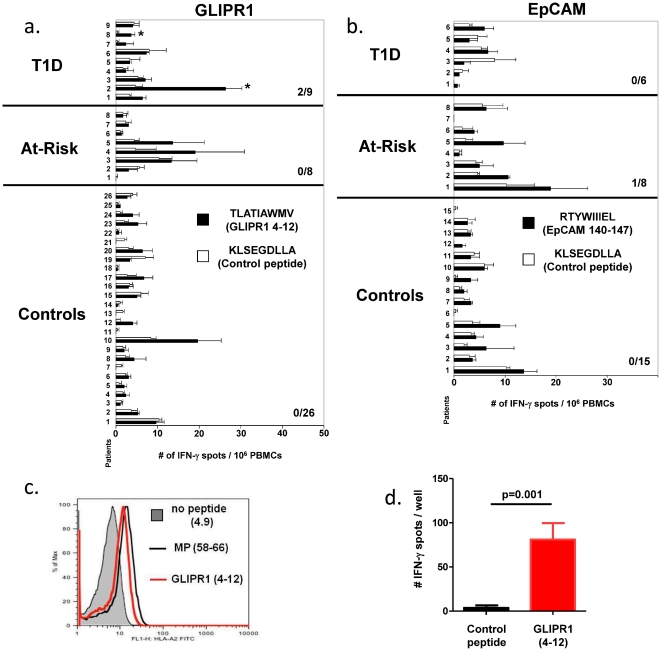
Responses to GLIPR1 4–12 and EpCAM 140–148 in cases and controls, and HLA restriction studies. Responses to **a**) GLIPR1 (4–12) **b**) and EpCAM (140–148) in T1D, at risk and control subjects. In both panels, PBMCs from the indicated patient groups were stimulated with GLIPR1 or EpCAM peptides (filled bars) vs. control peptide (open bars) overnight. Anti-CD3/CD28 stimulation was used as a positive control and all responses to antibody stimulation were too numerous to count (data not shown). The graphs display the average raw number of spots from triplicate wells +/− the standard error. The (*) indicates for a given patient that the experimental peptide responses were significantly higher (FDR <0.05) than the response to the control peptide. The number of patients who significantly responded to the each experimental peptide is indicated by the fraction in the graph. PBMC from subject ND2 used for discovery of each epitope were not available and are not included in these graphs. **c**) HLA-A*0201 binding assay for GLIPR1 epitope TLATIAWMV on T2 cells. 1×10^6^ T2 cells were incubated with 20 µg/ml of the indicated peptide in media for 4 hours at 37°C, stained for HLA-A*0201 and evaluated by flow cytomety. **d**). Presentation of GLIPR1 eptiope by HLA A*0201. T2 cells were loaded with 10 µg/ml GLIPR1 epitope TLATIAWMV or control epitope from pyruvate dehydrogenase for two hours at room temperature, washed and mixed with purified CD8+ T cells from one responding subject (T1D #2) in a direct IFNγ assay and was found to be statistically significant compared to the control peptide (FDR<0.05).

Because additional blood draws were unavailable from the subject used for discovery, we next tested overlapping 9 and 10 residue peptides from each reactive 15 residue peptide in multiple cases and controls. These studies identified a single epitope from each gene, TLATIAWMV for GLIPR1 (4–12), and RTYWIIIEL for EpCAM (140–148), as stimulating significant responses in newly diagnosed or at-risk subjects (an at-risk individual was defined as a person with a first degree relative with T1D having one autoantibody specific for a known T1D autoantigen) but rarely in controls (Fig. 3, panels a and b). Responses against GLIPR1 (4–12) were found to be significant in 2 of the 9 T1D cases; p-values 0.001 and 0.005 corresponding to FDR's of 0.011 (T1D#2) and 0.030 (T1D#8), respectively. One control individual (Control #12) out of 26 patients was also significant by p-value but it was not significant by FDR while no at-risk individuals were significantly different compared to the control peptide (Fig. 3a). There was no statistically significant response against EpCAM (140–148) in the T1D or control groups (Fig. 3b). Although, one at-risk subject had a significantly higher response against the indicated EpCAM peptide compared to a control peptide (at-risk #2) (p<0.05), this response was not significant by FDR. However, these data were generated without knowledge of the HLA class I alleles presenting each peptide eptiope. Assessing multiple cases and control subjects, all of which share the class I alleles presenting these peptides may reveal an elevated association with disease for GLIPR1 (4–12) and possibly EpCAM (140–148). Importantly, the consistent responses observed against minigene pools, individual minigenes and overlapping peptides in both cultured and direct ELIPOT assays for both GLIPR1 and EpCAM epitopes demonstrates that synthetic minigene screening identifies genuine, novel CD8+ T cell target epitopes.

Epitope prediction using BIMAS and SYFPEITHI suggested that the GLIPR1 epitope may be presented by HLA-A2 [17], [18]. We tested bothGLIPR1 and EpCAM epitopes for binding to and presentation by HLA-A*0201 using T2 cells [19], [20]. T2 cells are TAP deficient cells that solely express HLA-A*0201 and therefore have low levels of empty HLA-A*0201 on their surface. Loading T2 cells with exogenous peptide capable of binding HLA-A*0201 stabilizes the MHC class I molecule on the surface, leading to a detectable increase in HLA-class I surface staining. GLIPR1 (4–12), but not EpCAM (140–148) increased HLA-A*0201 surface staining, suggesting that GLIPR1 (4–12), but not EpCAM (140–148) can be presented by HLA-A*0201 (**Fig. 3c** **, and data not shown**). Furthermore, T2 cells loaded with GLIPR1 (4–12) were capable of stimulating IFNγ release by CD8+ T cells derived from responders identified in Figure 3A (**Fig. 3d**). Presentation of GLIPR1(4–12) by HLA-A*0201 is therefore sufficient for T cell recognition.

The subject used for discovery of EpCAM and the single at-risk subject with low but detectable responses to EpCAM 140–148 shared a single HLA class I allele, HLA-B*1501. Studies testing HLA-B*1501 for presentation of EpCAM 140–148 are ongoing. Interestingly, EpCAM is known to be upregulated during islet development and in many tumor types including insulinomas, suggesting that T cell responses against autoantigens induced during islet regeneration may be significant markers for disease progression [21], [22]. While both T cell and autoantibody responses against EpCAM have been reported in cancer patients, there have been no previous reports of immune responses against either EpCAM or GLIPR1 in subjects with type I diabetes [23]–[25], highlighting the ability of synthetic minigene screening to identify truly novel antigens.

## Discussion

Synthetic minigene screening can be immediately applied for CD8+ T cell antigen discovery in any system in which a rational selection of hundreds of candidate proteins, or tens of thousands of potential epitopes, can be made. In the example presented here, candidate antigens were chosen using expression array profiles to select genes exhibiting preferential expression in the target tissue, human islets [8]. However, a variety of other approaches to candidate antigen selection can be envisioned, including libraries selecting epitopes predicted to bind particular MHC alleles, peptides stripped from MHC and identified using mass spectrometry, or candidate minor antigenic peptides derived through genotyping of matched transplant donor and recipient pairs, i.e. candidate minor histocompatibility antigens. Indeed, libraries encoding the entire protein complement of pathogens with small genomes can be assembled with reagents and methodologies currently available.

Although programmable microarrays have previously been used to cost-effectively synthesize complex libraries of DNA and RNA species [15], to our knowledge this is the first study to use programmable microarrays to generate complex populations of polypeptides for discovery of T cell antigens. Although synthesis lengths remain limited, i.e. up to 200 bp, this length is sufficient to encode both polypeptides and supporting sequences for T cell antigen screening as described here (**Fig. 1**). The upper limit to the number of different proteins that can be screened is unknown but is not limited by the number of custom oligos that can be synthesized. Indeed, highly accurate libraries of as many as 55,000×150 bp oligonucleotides have been synthesized [9]. Experiments exploring larger minigene pool sizes are ongoing, and it is also possible that multiplex gene assembly [26], combinatorial pooling strategies [27], and optimized APC populations will increase the number of candidates that can be efficiently screened in the near future. In addition, minigene libraries directing synthesis of candidate peptides fused to autophagosomal targeting signals or secretory signals may allow discovery of CD4+ T cell epitopes [26], [28], [29].

One drawback to minigene screening is the current high cost and limited availability of long, accurate oligonucleotide libraries. However several vendors offer custom, complex oligonucleotide libraries for targeted sequence capture which may be suitable for minigene library construction. One critical parameter affecting the performance of minigene libraries is the error rate within the mixed oligonucleotide library template. While error rates in oligonucleotide mixtures synthesized on microarrays as low as 1 in 300 have been achieved, error rates of between 1 and 2% are common in libraries from several manufacturers, and include both deletions and single nucleotide substitutions. In some cases, non-uniform error rates have been observed, with a higher rate of errors near the 3′ end of sequences within a library versus the 5′ end. This may be due to the increased number of deblocking cycles experienced by the 3′ end of each oligonucleotide, resulting in a higher cumulative substitution rate near the solid support. Sequencing the amplified library with next generation sequencing technologies will be required to reveal the overall error rate and error distribution within this library. Application of enzymatic or MutS-based error correction strategies are expected to dramatically improve the accuracy of future libraries [30], [31]. Other than the libraries, the most costly reagents for synthetic minigene screening are the capped IVT kits, and the kits used for transfection of IVT products into APC. We estimate that a minigene screen of this scale costs approximately $10,000. However, a conservative estimate for synthesis of an equivalent overlapping peptide library would be in excess of $36,0000 (assuming $10/33 residue peptide×3669 peptides). Furthermore, the peptide library represents a finite resource, while a minigene library can be screened indefinitely for the additional cost of reagents for PCR, *in vitro* transcription and transfection.

A major outstanding question is the issue of false positive signals in the primary screen. It is interesting to note that each subject had multiple pools that scored positive in primary screens that were not clearly confirmed in initial deconvolution experiments, including two pools generating >100 spots in the primary screen. While experimental variation/error may have obscured deconvolution of pools with moderate spot numbers in cultured IFNγ ELISPOT screens (<30 spots), it might be expected that if genuine, the largest two responses would remain detectable in secondary assays. These results strongly suggest that false positive responses are occurring in the cultured ELISPOT screen albeit at an unknown frequency. This is not necessarily surprising, as ELISPOT assays are highly sensitive to a wide variety of factors [32]–[34]. In addition, the large number of individual tests performed in the primary screen may lead to detection of rare responses against materials/proteins derived from components of either the IVT reaction or the transfection solution itself. We are now performing experiments with purified IVT products, as well as with libraries in which each minigene is duplicated such that all possible pairs of pools have either one or zero minigenes in common. Duplications in this manner may reduce the number of false positive responses detected, while streamlining the deconvolution steps as well. Other possible variations in the protocol include using cell lines as APCs (such as K56A2 cells stably expressing HLA-A*0201 [35]) and eliminating the *in vitro* stimulation prior to the ELISPOT. This change may require use of greater numbers of CD8+ T cells/well (approx. 1–2×10^5^/well versus the 2×10^4^/well used here) for detection of low frequency CD8+ T cells. These modifications may ultimately reduce the number of false positives detected in the primary screen, thereby reducing the effort and costs associated with confirmation, deconvolution and validation.

High throughput-screening for novel T cell target antigens has been a major bottleneck for multiple subfields of immunological research, including cancer immunology, infectious disease, autoimmunity and transplantation. Initial studies presented here suggest that synthetic minigene library technology promises to overcome many of the difficulties associated with traditional screening techniques, immediately increasing the number of candidate T cell antigens amenable for screening from a handful of candidates to several hundred proteins or several thousand epitopes. We envision that extensions and improvements of this approach will enable comprehensive screens, i.e. all possible peptide epitopes represented in the full protein complement encoded by a pathogen or by a human genome.

## Supporting Information

Table S1 The Gene name, Affymetrix probeset number, percentile score (P), the entropy score (Q), P and Q ranking, and the number of tissues expressing the gene (N) are presented for each of 186 genes evaluated. The source column indicates whether the gene was evaluated by Wenzlau and Hutton (listed as H, reference 8), or was identified using a combination of P, N and Q scores (listed as P) as described in materials and methods, or was chosen as being co-expressed in islets and glomeruli (listed as G).
(XLS)

Table S2 Table S2 lists the nucleotide sequences of pool specific primers, common primer, minigene templates and primers used for assembly of the library.
(XLS)
